# The influence of simulated microgravity on MG-63 osteoblast-like cells cultured on polymeric scaffold

**DOI:** 10.1016/j.bbiosy.2026.100137

**Published:** 2026-04-17

**Authors:** Barbara Szaflarska, Kamila Walczak, Marcin Czepiel, Agata Kołodziejczyk, Elżbieta Pamuła

**Affiliations:** a AGH University of Kraków, Faculty of Space Technologies, al. Mickiewicza 30, 30-059 Kraków, Poland; b AGH University of Kraków, Faculty of Materials Science and Ceramics, al. Mickiewicza 30, 30-059 Kraków, Poland; c Jagiellonian University Medical College, Institute of Pediatrics, Department of Clinical Immunology, ul. Wielicka 265, 30-663 Kraków, Poland; d Analog Astronaut Training Center, Koło Strzelnicy 8A, 30-219 Kraków, Poland

**Keywords:** Microgravity, Tissue engineering, Bone tissue, Polymeric scaffolds, Plga

## Abstract

Bone degeneration remains a significant concern for long-term space missions due to microgravity-induced reductions in bone density and imbalances in bone remodeling processes. Discrepancies between spaceflight and ground-based analog studies complicate our understanding of the problem, particularly given the limitations of in vitro models that lack tissue-like environments. Research on osteoblast differentiation –crucial for bone maintenance – has yielded conflicting results, depending on the model and culture conditions. This study aims to establish a simple, scaffold-based bone tissue model using MG-63 cells cultured on poly(L-lactide-*co*-glycolide) (PLGA) scaffolds under simulated microgravity in random positioning machine (RPM), enabling further investigation into bone deconditioning mechanisms. After 14 days of culture, MG-63 cells exhibited osteogenic differentiation on both control flat glass substrate and PLGA scaffolds, with some variations between them, suggesting different maturation stages. Enclosure conditions in the vials, without contact with CO_2_ standard cell culture atmosphere, impaired cell differentiation, suggesting hypoxia-induced effects. Compared to the static standard cell culture counterparts, simulated microgravity tended to cause a decrease in expression of some osteogenic markers, such as runt-related gene 2 (Runx2) and type I collagen, and an increase in others, such as alkaline phosphatase (ALP). The use of scaffolds may modulate the effects of simulated microgravity. This simple bone tissue model shows potential for simulating microgravity-induced bone changes and could support research in Earth-based analog environments if proven in real microgravity.

## Highlights

• RPM device settings are crucial in providing adequate microgravity simulation.
• Microgravity and hypoxia can induce similar effects on MG-63 cells.
• Simulated microgravity changed the expression profile of MG-63 cells.
• The bone tissue model showed signs of deterioration in simulated microgravity.

## 1 Introduction

Bone deconditioning presents a major challenge to the safety of astronauts taking part in long-term space missions. Microgravity affects the skeletal system by reducing bone density and disrupting the balance between bone formation and resorption, inducing osteoporosis and significantly increasing the risk of fractures. These changes appear to be ongoing and progressive. Furthermore, exercise regimens alone have been shown to be insufficient to prevent them, highlighting the need for new pharmaceutical interventions [1,2]. Although changes in skeletal tissue in space have been studied extensively, some aspects remain poorly understood due to significant discrepancies between the findings from space-based studies and those conducted using various analogs and microgravity simulators [1,2]. In addition, most in vitro studies are conducted on isolated cells, which cannot fully reflect the behaviour of an actual tissue. Extracellular matrix (ECM), which plays an essential role in tissues in vivo, has been shown to take part in its reaction to changes in gravity, as cells can modify the expression patterns of ECM and cytoskeleton proteins in response to microgravity exposure [3]. However, very few studies have used the approach of incorporating a scaffold that can mimic the natural environment of bone tissue by providing appropriate mechanical and chemical signals [[4], [5], [6], [7]].

The most attention was paid to the osteoblast differentiation process, as these cells mediate the three crucial processes for bone health: osteogenesis, modelling, and remodelling [8]. The majority of studies on mesenchymal stem cells (MSCs) and preosteoblasts conducted in both real and simulated microgravity show that these conditions impede the differentiation of MSCs into osteoblasts. Exposure reduces the expression of osteoblastic gene markers and often leads the cells to undergo adipogenic differentiation instead [[9], [10], [11], [12], [13], [14], [15], [16]]. However, there are also studies that state otherwise and showed microgravity to enhance MSCs proliferation and osteogenic differentiation [[17], [18], [19], [20]].

As little as a day in microgravity simulated by a random positioning machine (RPM) resulted in the downregulation of osteogenic genes, such as runt-related gene 2 (Runx2), osterix, osteocalcin, collagen type I, and bone morphogenetic protein 1 (BMP1) in 2T3 preosteoblasts [12]. A week in a SJ-10 recoverable satellite caused human bone MSCs seeded on poly(lactic-*co*-glycolic acid) (PLGA) scaffolds to decrease the expression of 21 osteogenesis-related genes, among them alkaline phosphatase (ALP), collagen type I, and Runx2, instead promoting the expression of adipogenic markers [13]. The rodent hindlimb unloading (HLU) model [14] and the MSCs studies on polymeric microcarriers in simulated microgravity [11] showed similar outcomes. On the other hand, human MSCs differentiated into osteoblasts after 2 weeks of culture on the International Space Station (ISS) [17] as well as in simulated microgravity on the Zeromo [18], and on the RPM [20] devices. The MSCs originated from rats, cultured on calcium hydroxyapatite scaffolds in a 3D clinostat, resulted in suppression of the osteoblastic differentiation [21]. However, when studied on ceramic bovine scaffolds in a rotary wall vessel (RWV), they showed increased ALP levels and ability to repair bone defects in rat in vivo model after exposure to simulated microgravity [22].

MG-63 cells, frequently used as a pre-osteoblast model, showed reduced differentiation abilities and hindered the formation and maturation of ECM after 9 days of culture on coverslips on the Foton 10 satellite [23]*.* Exposure to simulated microgravity created with a RWV was found to decrease the growth of MG-63 cells and altered the structure of the cytoskeleton [24]. However, there are also reports from simulated microgravity, where MG-63 cells cultured on calcium phosphate-coated polycaprolactone nanofiber meshes increased the expression of the bone ECM proteins [25].

All of these non-coherent results emphasize the need for standardization in microgravity cell research. There is a wide variety of cell types, protocols, and simulators used, and it is clear that some differences between them and real microgravity cannot be avoided. Devices that simulate microgravity always exert an unwanted effect on the experiment, such as rotation-related forces like residual acceleration, movement of the cell medium, and shear forces [26,27]. Such influence should always be recognized and eliminated as much as possible by a thorough characterization of the device and cell model used. In that regard, there are still a lot of data missing, and further research is needed.

This study aims to create and assess a simple bone tissue model subjected to a simulated microgravity environment with the use of an RPM device that could reflect bone deconditioning experienced by astronauts and potentially allow high-throughput testing of active pharmaceutical ingredients if further characterized. After validation in real microgravity, such models may facilitate the development of systems for drug testing, which could be beneficial for research related to microgravity effects, as well as various health conditions on Earth. Microgravity has been demonstrated to mimic aspects of the aging process and related diseases [1,28,29], therefore might be helpful in modeling them. For simplicity, exposure to the RPM is referred to here as “simulated microgravity.” It should be noted, however, that, as discussed later in the manuscript, the RPM also introduces additional mechanical and environmental effects on the samples.

In our study, MG-63 osteoblast-like cells were chosen due to their robustness and phenotypic stability [30,31], while PLGA processed into a porous scaffold was selected to mimic the bone ECM environment due to a very good biocompatibility, porosity, and well-defined properties [32,33]. The total culture time was decided to be 14 days, as during that time osteogenic markers such as alkaline phosphatase (ALP), osteocalcin, and osteopontin can be detected in osteoblasts and the matrix development starts [34]. When induced by the presence of a biomaterial scaffold or osteogenic medium, these markers can also be found in MG-63 cells [[35], [36], [37]]. Although this cell line exhibits a less mature, pre-osteoblastic phenotype compared with primary osteoblasts, it remains a well-established and widely used model for preliminary investigations. Notably, its osteogenic potential can be enhanced, as it can upregulate bone-related markers when cultured in osteogenic medium or in the presence of biomaterials [31,38,39]. Additionally, the MG-63 cells show more resistance to biomaterial-induced cytotoxicity, similar to human bone MSCs, enhancing their predicted survivability in harsh simulated microgravity environment [31].

## 2 Materials and methods

### 2.1 Microgravity simulation

For microgravity simulation, a random positioning machine (RPM)/3D clinostat was used (provided by Analog Astronaut Training Center, Kraków, Poland). The device was previously characterized and tested with various biological systems [40,41]. The setting of ‘microgravity’ recommended by manufacturer is set to both inner and outer rings rotating at a speed of 60 rpm with random deviation. However, other researchers suggest that the optimal rotation speed for mammalian cells is 60 deg/s (10 rpm) [42]. According to Wuest et al., the centrifugal acceleration reaches an order of 10^–2^ G at a point 10 cm from the center of rotation at such moderate velocity and increases with both the velocity and the distance from the center of rotation [42]. To achieve the best microgravity simulation, both speeds were tested, 60 rpm in the first preliminary experiment, and 10 rpm in the second. The accelerations in the second experiment were estimated to be between 0.01 and 0.02 G (around 6 cm measured at the maximum distance from the center of rotation for any vial on the RPM) [42].

### 2.2 Materials and reagents

For scaffolds preparation, poly(L-lactide-*co*-glycolide) (PLGA) (molar ratio of L-lactide to glycolide 85:15, Mn = 100 kDa and *d* = 1.8, synthesized at the Polish Academy of Sciences, Zabrze, Poland) was used with sodium chloride (NaCl) (POCh, Gliwice, Poland) as a porogen and dichloromethane (DCM, 99.8 %, POCH Basic, Avantor Performance Materials Poland S.A.) as a solvent. The scaffolds were modified with collagen (type I, human recombinant, expressed in *Nicotiana tabacum*, stock concentration 2.5–3.5 mg/ml, Sigma-Aldrich, CollageTM, CollPlant Ltd.). Optical microscopy images of the scaffolds were taken using a Stereo Discovery V8 microscope (Carl Zeiss, Germany) and scanning electron microscopy (SEM, NOVA NANO SEM 200) in the low-vacuum conditions. Scaffolds were weighted using a Balance XSE105DU (Mettler-Toledo, Poland) laboratory scale.

MG-63 cells (European Collection of Cell Cultures, Salisbury) were used in the experiments. All cell culture media and supplements were purchased from PAN-Biotech, except for non-essential amino acids (NEAA), sodium pyruvate, and (4-(2-hydroxyethyl)-1-piperazineethanesulfonic acid) (HEPES) solution, which was purchased from ThermoFisher. Chemical reagents such as Triton X-100, formaldehyde, ALP buffer and NaCl were obtained from Sigma Aldrich, and HCl was obtained from POCh. Phosphate buffered saline (PBS) was obtained from VWR. Well plates were purchased from SARSTEDT and polypropylene vials from FL Medical. The pH was measured with a CP-401 pH-meter (Elmetron).

Cell culture evaluation was performed using AlamarBlue Cell Viability test (ThermoFisher), lactate dehydrogenase activity assay (LDH, Takara, Saint-Germain-en-Laye, France), ALP activity assay based on p-nitrophenol (Sigma Aldrich), calcein-AM and propidium iodide (PI) staining (Sigma Aldrich), Alexa Fluor 488 phalloidin and 4′,6-diamidino-2-phenylindole, dihydrochloride (DAPI) staining (ThermoFisher). For absorbance measurements, a fluorometer (FluoStar OMEGA, BMG LabTech) was used and the imaging was performed on an AxioVert 40 fluorescence microscope (Carl Zeiss, Germany). The RT-qPCR was performed with the use of the following reagents: RNAlater Stabilization Solution (ThermoFisher), RNeasy Mini Kit (Qiagen), High-Capacity cDNA Reverse Transcription Kit (ThermoFisher), and Fast SG qPCR Master Mix (2x) plus ROX solution (EURX). The RNA concentration was measured with a NanoDrop One spectrophotometer (ThermoFisher) and the reaction was carried out on a QuantStudio 6 Flex Real-Time PCR System (ThermoFisher).

### 2.3 Preparation of the scaffolds

Scaffolds were made from PLGA using the solvent casting/porogen leaching method according to a procedure described previously [43]. Sieved NaCl particles with grain diameter between 250–320 µm were used as porogen in a volume fraction of 85 %, as those parameters were previously shown to be within range for effective osteoblastic cell colonization [43,44]. DCM was used as a PLGA solvent. In brief, PLGA was dissolved in DCM and mixed with NaCl in Petri dishes until most of the solvent evaporated. Afterwards, the obtained paste was packed into polypropylene syringes and cut into slices after complete evaporation of DCM. To remove the porogen, scaffolds were washed in MilliQ water. The size of the scaffolds were: diameter 12 mm, thickness 1.5 mm, and weight 20 ± 1 mg in the first preliminary experiment (big scaffolds) and: diameter 8.5 mm, thickness 1.5 mm and weight 10 ± 1 mg in the second experiment (small scaffolds). The cell number and medium volume were scaled accordingly to keep the same material mass: medium volume: cell number ratio.

Before the experiments, the scaffolds were sterilized by immersion in 70 % ethanol for 20 min and exposure to UV light for another 20 min per each side. Glass coverslips were autoclaved.

In the first preliminary experiment (60 rpm), scaffolds were modified with collagen by soaking in 40 µg/ml collagen in PBS solution, according to a previously described method [45]. First, the air was removed from the pores by creating a vacuum with a syringe, and subsequently, the scaffolds were left in the solution for 14 h. In the second experiment, the lower rotation speed (10 rpm) was applied and the samples were not coated with collagen. In both experiments, glass coverslip samples served as a biocompatible control material.

### 2.4 Characterization of the scaffolds

Scaffolds were characterized by optical microscopy, SEM, and X-ray computed nanotomography. On the basis of the optical microscopy images, average pore size and shape factor, defined as the ratio of the largest size value to perpendicular size value, were calculated. Scaffolds were weighted and their thickness was measured using a caliper. X-ray computed nanotomography was applied to analyze the internal structure of scaffolds. Experiment was performed on a GE (Phoenix) Nanotome S high-resolution computed nanotomography system. Measurements were made at a voltage of 60 kV and an X-ray tube current of 200 µA. A total of 2400 projections were acquired with a single exposure time of 0.5 s. Each projection was averaged from four images. The final voxel size was 1.5 µm. The tomograms were registered using a Hamamatsu 2300 × 2300 pixel 2D detector. The reconstruction of the measured objects was performed using datosX ver. 2.1.0 (GE) with the use of a Feldkamp algorithm for cone beam X-ray CT [46]. In this case, extensive scaffold characterization was not performed, as materials manufactured from the same polymer and with the same method have been thoroughly studied in the past, achieving porosity of 85 % [32,33,37].

### 2.5 Cell culture

**Fig. 1:**
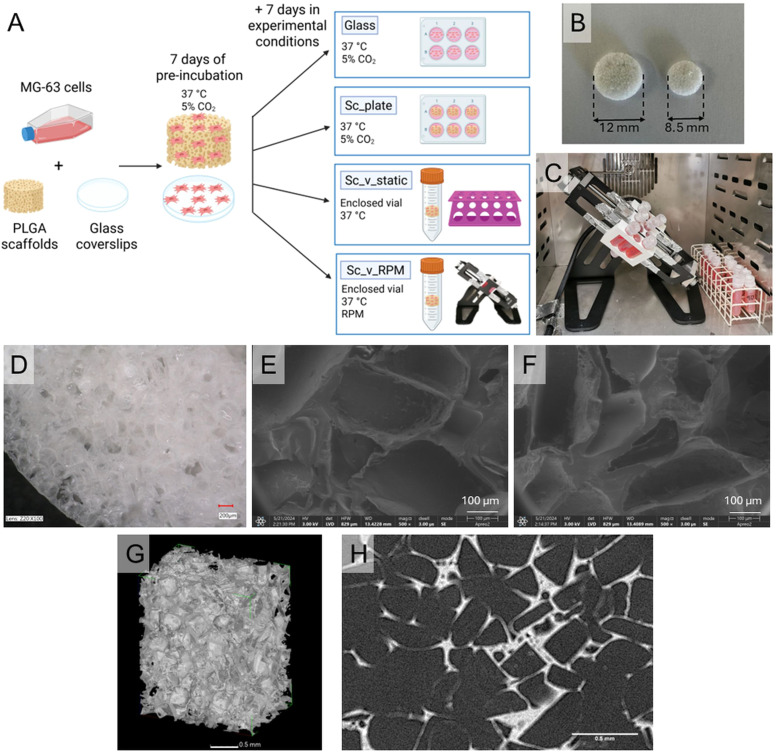
(A) Schematic representation of experimental design. MG-63 cells were cultured either on scaffolds or glass coverslips at 37 °C in 5 % CO_2_ atmosphere for 7 days (pre-incubation). Then cells on glass coverslips (Glass) and scaffolds (*Sc*_plate) were cultured at 37 °C in 5 % CO_2_ atmosphere for another 7 days as controls. Cells were additionally cultured for another 7 days on scaffolds without contact with 5 % CO_2_ atmosphere in enclosed vials at 37 °C (*Sc*_v_static) and in enclosed vials submitted to RPM (*Sc*_v_RPM). (B) For experiment 1 bigger scaffolds (12 mm in diameter) were used and seeded with 50,000 cells, while for experiment 2 smaller scaffolds (8.5 mm in diameter) were used and seeded with 25,000 cells. (C) Testing conditions for *Sc*_v_RPM (left) and *Sc*_v_static (right). (D) Optical microscopy picture of the scaffold. Scanning electron microscopy (SEM) pictures of: (E) non-modified poly(L-lactide-*co*-glycolide) (PLGA) scaffold, (F) collagen-modified PLGA scaffold. X-ray computed nanotomography images of PLGA non-modified scaffolds: (G) 3D reconstruction and (H) 2D projection.

Before MG-63 cell seeding, scaffolds were pre-wetted with medium by exposing them to low-pressure conditions, according to a method described previously [47]. MG-63 osteoblast-like cells were seeded on the scaffolds with a density of 50,000 cells / big scaffold and 25,000 cells / small scaffold and on glass coverslips with a density of 5 500 cells/cm^2^. All samples were pre-cultured for 7 days in standard conditions (37 °C, 5 % CO_2_), allowing the cells to adhere and proliferate on the materials. After that time, scaffold samples were divided into three groups: (1) scaffolds placed in the 24-multiwell plate in 2 ml of medium and cultured in the cell incubator under standard cell culture conditions (37 °C, 5 % CO_2_) (*Sc*_plate), (2) scaffolds in the enclosed vials (9 ml / big scaffold and 4.5 ml / small scaffold) fully filled with medium, placed in the incubator (37 °C) without contact with CO_2_ (*Sc*_v_static) and (3) scaffolds in the enclosed vials fully filled with medium placed in the RPM device (60 rpm for the first experiment and 10 rpm for the second experiment) in the incubator (37 °C) without contact with CO_2_ (*Sc*_v_RPM). Glass control samples were placed in a 24-well plate in the incubator under standard conditions (37 °C, 5 % CO_2_) (Fig. 1). Cells were cultured in Eagle Minimum Essential Medium (EMEM) with l-glutamine and 1.5 g/l NaHCO_3_, supplemented with 10 % fetal bovine serum (FBS), 1 % of penicillin and streptomycin, 0.1 % NEAA, and 0.1 % sodium pyruvate. Moreover, in the second experiment, the medium in all samples was supplemented with 25 mM HEPES to increase the buffering capacity in the enclosed vials, and for *Sc*_v_RPM the device was set at 10 rpm. During the experiments, the medium in the well plates was exchanged every three days, while the enclosed samples in the vials were kept unopened. After 7 days of culturing under experimental conditions, the RPM was stopped and the samples were taken for evaluation. The medium pH was measured shortly after opening the vials with the use of a pH-meter. A summary of protocol optimization between the first preliminary experiment and the second experiment is included in supplementary materials (Table 1S).

### 2.6 Cell viability assay

Cell viability was assessed using the AlamarBlue Cell Viability test, based on resazurin reduction (*n* = 5 per experiment). The medium in the tested samples was removed and replaced with a 10 % AlamarBlue reagent solution in EMEM (1 ml per well). The samples were then incubated under standard conditions (37 °C and 5 % CO_2_). After incubation, 150 µl of the solution was aspired and placed in a black 96-well plate, with three replicates for each sample. The absorbance was measured with a fluorometer at excitation wavelength 544 nm and emission wavelength 590 nm. The results are expressed as the % of resazurin reduction calculated as the ratio of a sample measurement and a 100 % reduced sample, both net of a blank sample, according to a formula reported earlier [48].

### 2.7 Cytotoxicity assay and ALP activity assay

For cytotoxicity assessment, the LDH activity assay was applied (*n* = 3 per experiment). LDH working solution was prepared from reagent A (INT/sodium lactate) and reagent B (catalyst (A) diaphoresis/NAD). The assay was conducted on both supernatants and cell lysates. For supernatants, the medium was collected and placed in a 96-well plate, 50 µl per well, with three replicates for each sample. A sample of fresh cell culture medium was also tested. For lysates, the medium was removed and 400 µl of 1 % Triton X-100 was added to each sample. Samples were put on a shaker for 50 min, after which the lysate was collected and placed in a 96-well plate, 50 µl per well, with three replicates for each sample. 50 µl of the LDH working solution was added to each well in both supernatant and lysate plates. The plates were incubated in darkness for 8 min, followed by the addition of the LDH stop solution (0.5 M HCl). The absorbance was measured (wavelength 492 nm) with a fluorometer. The results are presented as cytotoxicity in %, calculated as the ratio of the supernatant and the lysate measurements.

In the second experiment (10 rpm), ALP activity was also measured. A tablet of p-nitrophenol was added to 20 ml of ALP buffer (pH = 9.8) to prepare the working solution. From the lysed samples, 25 µl of the lysate was collected and placed in a 96-well plate, with three replicates for each sample. The plate was kept on ice, while 125 µl of the ALP working solution was added to each well. The plate was then incubated at 37 °C for 30 min, followed by the addition of the ALP stop solution (1 M NaOH). The absorbance was measured (wavelength 405 nm) with a fluorometer. Results are presented as the ratio of ALP and LDH lysate measurements.

### 2.8 Fluorescence microscopy

Live/dead staining with calcein-AM and PI was performed in both experiments to visualize viable and dead cells (*n* = 2 per experiment). In the second experiment (10 rpm), phalloidin and DAPI staining were also applied in order to observe the actin cytoskeleton structure and the condition of the cell nuclei (*n* = 2). Microscopic observations were made using a fluorescence microscope.

#### 2.8.1 Calcein-AM and PI staining

A staining solution was prepared by mixing calcein-AM, PI, and PBS in the proportions of 1:1:1000. Medium was removed from the wells with tested samples and replaced with 1 ml of staining solution per well. The samples were then incubated in darkness for 15 min.

#### 2.8.2 Phalloidin and DAPI staining

The phalloidin and DAPI staining was conducted exclusively during the second experiment. The samples were first fixed with 3.7 % formaldehyde solution The cell medium was removed, the samples were washed with PBS enriched with magnesium and calcium ions, and the formaldehyde solution was added. After 20 min, the solution was replaced with a fresh one and the samples were kept at 4 °C prior to evaluation. On the day of staining, formaldehyde solution was removed, samples were washed two times with PBS enriched with magnesium and calcium ions, and submerged in a 0.1 % Triton X-100 solution for 15 min. After that, the samples were washed once again two times with PBS enriched with magnesium and calcium ions.

Phalloidin working solution was prepared by diluting 1.125 µl of the Alexa Fluor 488 phalloidin stock solution (1 vial in 150 µl of DMSO) in 450 µl of PBS per sample. To completely submerge the scaffolds, 450 µl of phalloidin working solution was added to each well with a sample. The plate was incubated at room temperature for 60 min. DAPI working solution was prepared by diluting the DAPI stock solution (1 mg/ml) in PBS in the proportions of 1:1000. After removing the phalloidin working solution, DAPI working solution was added (450 µl per well) and the plate was incubated in darkness for 10 min.

### 2.9 RT-qPCR

RT-qPCR was conducted exclusively during the second experiment (*n* = 3). Following sample collection, each well received 450 µl of RNAlater stabilization solution for RNA preservation. The samples were stored at −80 °C for several days prior to processing. RNA isolation was performed with the RNeasy Mini Kit, according to the manufacturer’s instructions. After adding 600 µl of RLT buffer, the scaffolds were homogenized by mechanically disrupting the structure with a pipette tip. Subsequent steps were performed according to the instructions provided by the manufacturer. RNA concentration was determined using a NanoDrop One spectrophotometer. Reverse transcription (RT) was performed using the High-Capacity cDNA Reverse Transcription Kit with 5 µl of extracted RNA. The selected primers included fibronectin 1 (Fib1), Runx2, ALP, collagen type I (Col1), osteopontin (SPP1), osteocalcin (BGLAP), and GAPDH as a reference gene (primer sequences is shown in supplementary materials, Table S2). Real-time qPCR was conducted with Fast SG qPCR Master Mix (2x) plus ROX solution on the QuantStudio 6 Flex Real-Time PCR System using a 2-step cycling protocol for 40 cycles. The results were analysed using the standard ddCt method [49] and presented as relative expression compared to control conditions (Glass samples).

### 2.10 Statistical analysis

Statistical analysis and plot generation were performed using the Origin software (OriginLab Corporation). Quantitative results are presented as an average ± standard deviation. The Kruskal-Wallis nonparametric test was applied for sample comparison with probability values lower than 0.05 (p* < 0.05) considered statistically significant. Significance levels are indicated at p* < 0.05; p** < 0.01; p*** < 0.001.

## 3 Results

Materials characterization was performed before starting the in vitro experiments. All the cell culture assays were performed after 7 days of pre-incubation in standard conditions and 7 days in experimental conditions (*Sc*_v_static, *Sc*_v_RPM) or standard conditions (*Sc*_plate, Glass) as reference.

### 3.1 Scaffolds characterization

The pores in the scaffolds were mostly round or slightly elongated. Average pore size, measured as substitute diameter, was 260 ± 80 μm and average shape factor ranged between 1.0 and 1.4. The optical microscopy picture is presented in Fig. 1D The pore size histogram is included in supplementary material (Fig. 1S). Pores were confirmed to be of the same characteristics inside the scaffold as on the surface by X-ray computed nanotomography images (Fig. 1G and H). The distribution of the pores throughout the scaffold was uniform.

Scaffolds modified with human recombinant collagen (Fig. 1F) showed no structural differences as compared to those non-modified (Fig. 1E), as illustrated by SEM images. Modification was performed to increase the adhesion of the cells to the scaffold material. However, no significant differences were observed in cell adhesion between nonmodified and collagen modified and scaffolds, contrary to other studies [45]. Therefore, this modification was not performed for the second experiment (10 rpm).

### 3.2 Cell viability, cytotoxicity, ALP activity and medium pH values

**Fig. 2:**
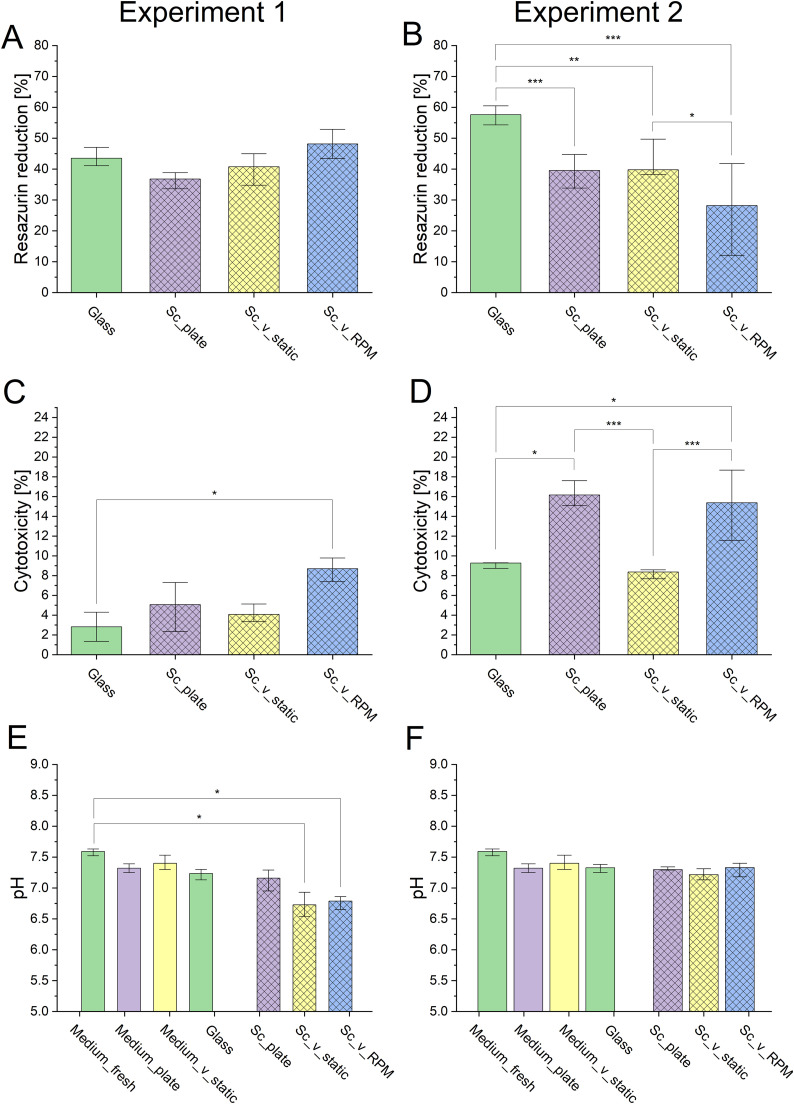
Results of experimental conditions on MG-63 cell culture in Experiment 1 (A, C, E) and Experiment 2 (B, D, F): (A, B) AlamarBlue resazurin reduction, (C, D) LDH assay, (E, F) pH value of medium after cell culture. Sample description: Medium_fresh – cell culture medium without samples, Medium_plate – cell culture medium without samples kept in the well plates in standard conditions (37 °C, 5 % CO_2_), Medium_v_static – cell culture medium without samples enclosed in the vials (37 °C), Glass – cells seeded on glass coverslips and cultured in standard conditions (37 °C, 5 % CO_2_), *Sc*_plate – cells seeded on scaffolds in the well plates and cultured in standard conditions (37 °C, 5 % CO_2_), *Sc*_v_static – cells seeded on scaffolds, enclosed in the vials (37 °C), *Sc*_v_RPM – cells seeded on scaffolds, enclosed in the vials (37 °C), and subjected to the 60 rpm (Experiment 1) and 10 rpm (Experiment 2) settings on the RPM. Statistical significance p* < 0.05; p** < 0.01; p*** < 0.001 according to Kruskal-Wallis nonparametric test.

In the first preliminary experiment after 14 days of culture cells did not show any significant changes in the resazurin reduction and even showed the tendency to increase their metabolic activity when cultured in enclosed vials without contact with CO_2_ atmosphere (*Sc*_v_static) and additionally submitted to 60 rpm setting on the RPM (*Sc*_v_RPM) in comparison to cells cultured in control conditions, i.e. in the incubator with CO_2_ atmosphere (*Sc*_plate) (Fig. 2A). In the second experiment (Fig. 2B), the observed effect was opposite – cells significantly lowered their activity in response to the 10 rpm setting on the RPM as compared to control (*Sc*_plate) and static (*Sc*_v_static) conditions. Resazurin reduction did not change significantly in enclosed vials (*Sc*_v_static) compared to the plate controls, however, it decreased in RPM conditions (*Sc*_v_RPM) compared to static samples, suggesting that solely RPM conditions were responsible for this change.

For Experiment 2, cytotoxicity assessed by LDH assay was twofold higher for all samples as compared to Experiment 1 (Fig. 2D vs Fig. 2C), but the samples exhibited a similar pattern. RPM conditions at 60 rpm (Fig. 2C) and 10 rpm (Fig. 2D) were more cytotoxic as compared to the conditions when cells were cultured on glass coverslips. Surprisingly, for Experiment 2 cytotoxicity was also increased for cells cultured in the plates stored in the CO_2_ incubator, as compared to glass coverslips and *Sc*_v_static, i.e. in the closed vials without the contact with CO_2_ atmosphere. Presumably addition of HEPES was beneficial in reducing cytotoxicity of *Sc*_v_static. There was also a significant increase in cytotoxicity in *Sc*_v_RPM samples compared to the static counterparts. Nonetheless, the cytotoxicity level in all samples was acceptable and did not significantly affect the culture, which was also confirmed by the live/dead staining assay. A slight cytotoxicity level increase in response to the scaffolds was expected, as foreign materials can induce a cellular response.

In Experiment 1, a drop in cell medium pH level was observed in the enclosed samples (*Sc*_v_static and *Sc*_v_RPM) compared to fresh medium (Fig. 2E). To avoid the pH drop in the second experiment, HEPES was added to the medium in order to increase the buffering capacity. As shown in Fig. 2F, in the second experiment the pH was at a similar level in all of the tested samples.

**Fig. 3:**
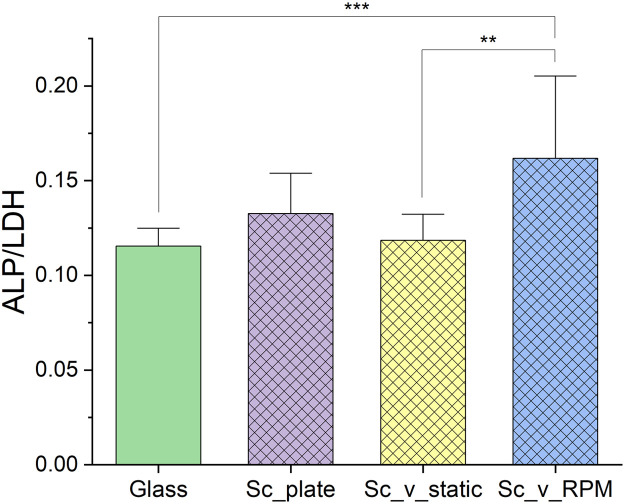
ALP activity of the cells in Experiment 2. Sample descriptions: Glass – cells seeded on glass coverslips and cultured in standard conditions (37 °C, 5 % CO_2_), *Sc*_plate – cells seeded on scaffolds in the well plates and cultured in standard conditions (37 °C, 5 % CO_2_), *Sc*_v_static – cells seeded on scaffolds, enclosed in the vials (37 °C), *Sc*_v_RPM – cells seeded on scaffolds, enclosed in the vials (37 °C), and subjected to the 10 rpm setting on the RPM. Statistical significance p* < 0.05; p** < 0.01; p*** < 0.001 according to Kruskal-Wallis nonparametric test.

ALP activity, which is an early osteogenic differentiation marker, appeared to be relatively similar among all the samples (Fig. 3), rising slightly in the *Sc*_plate samples. In RPM samples, the ALP activity was significantly higher compared to the samples in static conditions and the glass control, indicating that the ALP increase was caused by the RPM exposure.

### 3.3 Live/dead staining and cell distribution within the scaffolds

**Fig. 4:**
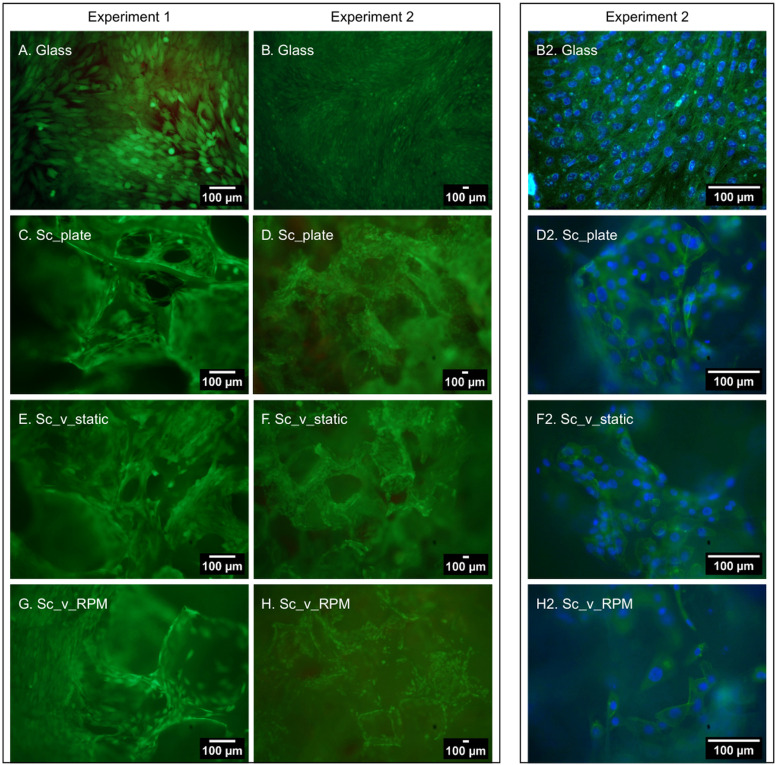
Live/dead staining results in Experiment 1 (A, C, E, G) and Experiment 2 (B, D, F, H) of MG-63 cells cultured on (A, B) Glass, (C, D) *Sc*_plate, (E, F) *Sc*_v_static, (G, H) *Sc*_v_RPM. Living cells stained green by calcein-AM, dead cells stained red by propidium iodide. Phalloidin/DAPI staining results in Experiment 2: (B2) Glass, (D2) *Sc*_plate, (F2) *Sc*_v_static, (H2) *Sc*_v_RPM. Actin filaments stained green by phalloidin, cell nuclei stained blue by DAPI. Glass – cells seeded on glass coverslips and cultured in standard conditions (37 °C, 5 % CO_2_), *Sc*_plate – cells seeded on scaffolds in the well plates and cultured in standard conditions (37 °C, 5 % CO_2_), *Sc*_v_static – cells seeded on scaffolds, enclosed in the vials (37 °C), *Sc*_v_RPM – cells seeded on scaffolds, enclosed in the vials (37 °C), and subjected to the 60 rpm (Experiment 1) and 10 rpm (Experiment 2) settings on the RPM. Scale bar =100 µm.

In both Experiments 1 and 2, live/dead staining showed elongated cells stained green in high density in the control glass samples, with very few dead cells stained red, suggesting that they almost reached maximum confluence (Fig. 4A and B). No significant changes in morphology were observed in any of the experimental samples, and isolated dead cells were observed deeper inside the pores of the scaffold regardless of the conditions. In the control (*Sc*_plate) and samples cultured in static enclosed conditions (*Sc*_v_static), it was easy to find large clusters of cells on the surface of the material, spreading on the pore walls (Fig. 4C-F). In the samples exposed to RPM conditions (*Sc*_v_RPM), those clusters were harder to find, suggesting either lower cell number or successful migration of the cells deeper inside the scaffold caused by the experimental conditions (Fig. 4G and H). On the basis of images of deeper focal planes, cells under RPM conditions appeared to be located deeper within the scaffold than static samples. However, this observation is qualitative and is not supported by quantitative analysis of the entire scaffold volume.

### 3.4 Phalloidin and DAPI staining

Phalloidin and DAPI staining confirmed the results of the live/dead staining, showing well spread, elongated cells on control glass coverslips (Fig. 4B2). It also showed more visible cell clusters on the surface of *Sc*_plate and static scaffold samples than on the ones exposed to RPM conditions (Fig. 4 D2, F2, and H2, respectively). Actin cytoskeleton visualization confirmed that cells adhered well to the materials and were found inside the pores of the scaffolds. All observed cells were mononucleated regardless of condition and the nuclei did not show any signs of irregularities. In the samples exposed to RPM conditions, a few cells with fragmented nuclei were spotted, suggesting that apoptosis could be a possible mechanism explaining the increased cytotoxicity level. However, it must be noted that these cells constituted a very small fraction of the total number of the observed cells.

### 3.5 RT-qPCR

The genes for RT-qPCR were chosen based on the three processes of normal bone development: (1) proliferation and ECM synthesis, characterized by type I collagen expression, (2) ECM development and maturation, characterized by ALP expression immediately after the proliferative phase, and (3) ECM mineralization, characterized by osteocalcin (BGLAP) and osteopontin (SPP1) expression [34]. Apart from those, the expression of Runx2, as the main osteogenic transcription factor, and fibronectin I, as a protein involved in cell adhesion and a component of the ECM, were assessed.

There were no statistically significant differences between the cells cultured on glass coverslips (Glass) and cells cultured on scaffolds in standard conditions (*Sc*_plate), however, a tendency for a different gene expression profile can be observed with increased Runx2 and ALP and decreased type I collagen, SSP1, and BGLAP expression in *Sc*_plate samples.

**Fig. 5:**
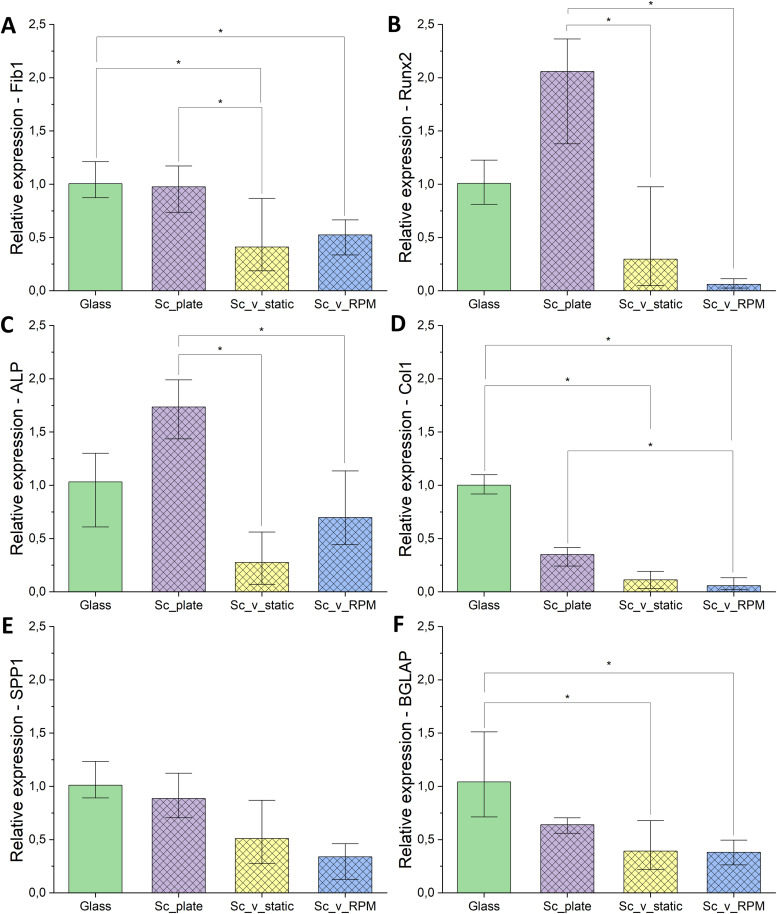
RT-qPCR results in Experiment 2: (A) Fib1 – fibronectin 1, (B) Runx2 – cbfa1, (C) ALP – alkaline phosphatase, (D) Col1 – collagen type I, (*E) spp1* - osteopontin, (F) BGLAP – osteocalcin. Glass – cells seeded on glass coverslips and cultured in standard conditions (37 °C, 5 % CO_2_), *Sc*_plate – cells seeded on scaffolds in the well plates and cultured in standard conditions (37 °C, 5 % CO_2_), *Sc*_v_static – cells seeded on scaffolds, enclosed in the vials (37 °C), *Sc*_v_RPM – cells seeded on scaffolds, enclosed in the vials (37 °C), and subjected to the 10 rpm setting on the RPM. Statistical significance p* < 0.05; p** < 0.01; p*** < 0.001 according to Kruskal-Wallis nonparametric test.

Cell culture on the scaffolds in the vials (*Sc*_v_static) caused a decrease in expression of all of the assessed genes as compared to cell culture in standard conditions in the CO_2_ incubator (*Sc*_plate), with significant differences in fibronectin I, Runx2, and ALP (Fig. 5A, B, C, respectively). A tendency to decreased collagen type I, SPP1, and BGLAP expression level can also be observed, although the results are not statistically significant (Fig. 5D, E, F, respectively).

Statistically significant differences were not found between the cells cultured on the scaffolds in the enclosed vials in static conditions and submitted to the 10 rpm setting on the RPM, although relative expression of Runx2 and Col1 in the latter was extremely low (Fig. 5B, D, respectively), while expression of ALP and fibronectin (Fig. 5C, A) tended to be higher.

## 4 Discussion

Conditions imposed on cell cultures by the RPM equipment are complex and require careful analysis. While simulated microgravity is the primary intended stimulus exerted on the samples, other factors – such as residual accelerations and hypoxic conditions – must also be considered when designing experiments and interpreting obtained results. Therefore, in the experimental setup described, ‘RPM conditions’ encompass the combined effects of simulated microgravity, hypoxic environment, and residual accelerations. Such limitations are imposed by the device’s working principles. As the residual accelerations grow with the distance from the axis of rotation, the sample containers must be as small as possible. Moreover, the rotating system requires the containers to be liquid- and gas-tight to eliminate spills and bubbles forming inside. To balance the device’s limitations and accurate simulations of microgravity conditions, small, enclosed vials were used as sample containers. To distinguish the specific impact of simulated microgravity from hypoxic effects, comparisons were made between MG-63 cells cultured under typical static conditions (37 °C, 5 % CO_2_) on both flat glass substrate and on PLGA scaffolds, as well as on PLGA scaffolds enclosed in vials filled with medium (37 °C, without CO_2_) (*Sc*_v_static) and the latter submitted to RPM (*Sc*_v_RPM). However, it would be worth for future research to also measure hypoxia in the enclosed samples.

Furthermore, it is essential to select the device settings carefully. The two rotational speeds at 60 rpm in Experiment 1 (the factory setting for microgravity simulation) and 10 rpm in Experiment 2 yielded different outcomes. The higher speed of 60 rpm introduced greater residual accelerations on the samples, which may have contributed to increased cellular activity, as shear forces are known to promote activity in bone cells [[50], [51], [52]]. Conversely, the speed of 10 rpm was hypothesized to have created a state more closely resembling microgravity by introducing more non-disturbed free-fall conditions. As the majority of studies on various bone cells showed their decreased proliferation and activity in microgravity [2,13,23,24], the speed of 10 rpm, better mimicking those results, was chosen for further evaluation. In Experiment 1, there was no significant difference in cell viability, whereas at 10 rpm in Experiment 2, cell viability was reduced and cytotoxicity level was increased, which is in accordance with the literature data. Although it must be kept in mind that the cytotoxicity assay result might also be caused by supplementing the medium with HEPES buffer, which indeed stabilized pH of the culture medium, but might exert some toxic effects [53].

The discrepancy between the RPM’s factory setting of simulated microgravity (60 rpm) and that suggested by the literature (10 rpm) highlights the current lack of standardization in microgravity cell culture research. It is important to note that the simulation of microgravity should be evaluated on a case-by-case basis, and adjustments to the system may be necessary. The factory settings of the described RPM may be more appropriate for other biological systems, such as plants, rather than for cell culture applications [54].

According to Stein et al., after 14 days of culture, the differentiation of the osteogenically induced bone cells should reach the ECM maturation phase, characterized by elevated ALP expression. The type I collagen levels may begin to decline but should remain relatively high, and the osteocalcin and osteopontin expression might start rising [34]. In this experiment, the material used for cell culture was treated as an osteogenic inducer, therefore expression of the aforementioned genes was expected on both materials. Notably, even though MG-63 cell line is considered pre-osteoblastic, after 14 days of culture, the expression of bone markers can be similar to primary osteoblasts, except for collagen type I [38].

All tested mRNA transcripts were detected in all samples, suggesting that the MG-63 osteoblast-like cells are at the osteogenic differentiation process both on glass coverslips and PLGA scaffolds. Cells seeded on PLGA scaffolds (*Sc*_plate) exhibited a tendency for higher levels of Runx2 and ALP expression compared to those seeded on glass coverslips (Glass). Conversely, the expression of type I collagen, SSP1, and BGLAP was reduced (although not significantly), suggesting that the cells were at different stages of differentiation in each condition. A successful differentiation can be further supported by detection of ALP activity, which is normally not present in MG-63 cells cultured in a monolayer [38].

Cells on the PLGA scaffolds both in the enclosed vials (*Sc*_v_static) and submitted to simulated microgravity (*Sc*_v_RPM) showed impaired osteogenic differentiation compared to the controls in the well plate (*Sc*_plate), as indicated by decreased expression levels of all the examined mRNAs. This result can be explained by the hypoxic conditions that were inevitably created by the enclosed vials over the 7 days of the experiment. Hypoxia is a well-documented factor that can inhibit bone growth and differentiation [[55], [56], [57], [58], [59]]. It is also one of the factors that can occur in bone unloading and it has been shown to cause a decrease in Runx2, ALP, collagen type I, and BGLAP expression in osteoblasts [55,58,59] and MG-63 osteoblast-like cells [56]. Presented findings are consistent with previously reported effects of hypoxia on MG-63 cells [56]. Notably, enclosure resulted in a significant decrease in ALP mRNA levels, although the corresponding reduction in ALP activity was small and not statistically significant.

Cells cultured under simulated microgravity conditions (*Sc*_v_RPM) compared to those cultured statically (*Sc*_v_static) exhibited slightly lowered expression of genes such as Runx2, type I collagen, and osteopontin, suggesting impaired differentiation, and slightly increased expression of ALP and fibronectin, although the results were not statistically significant. However, the ALP activity levels were significantly increased in those samples, suggesting that conditions created by the RPM may have promoted the early osteogenic differentiation of those cells.

MG-63 cells have previously been reported to exhibit a decrease in the expression of osteoblastic differentiation-related genes under both real and simulated microgravity conditions [23,24]. However, there were also experiments showing increased differentiation after simulated microgravity exposure [25]. Araujo et al. seeded MG-63 cells onto nanofibre meshes and hypothesized that the presence of a scaffold might enhance the beneficial effects that an RWV can have on bone cell cultures. This effect could be attributed to the shear stress and accelerations induced by the RWV, alternatively, it is also possible that the biomaterial scaffold alleviates or modifies the adverse effects of microgravity on bone tissue. A similar mechanism might explain the results of the described experiment, although it should be noted that, to the authors’ knowledge, this was the first time that this model of RPM was applied to scaffold-supported bone cell culture research and its effects might differ from those observed with the previously described RWV. Nevertheless, we can hypothesize that the micro-environment inside the pores of the PLGA scaffold decreased the influence of the simulated microgravity on the cells. A similar effect was reported by Avitabile et al. in a study on human MSCs where the magnesium-doped hydroxyapatite/type I collagen composite scaffold alleviated the RPM-induced osteogenic differentiation impairment [7].

## 5 Conclusions

The described work constituted two experiments that focused on assessing a simple bone tissue model in a simulated microgravity environment. Their results showcase the importance of careful adjustment of the experimental conditions when working with microgravity simulators. In the first preliminary experiment, the collagen modification did not result in increased cell adhesion and setting the RPM at 60 rpm did not significantly change cell viability or cytotoxicity.

In the second experiment, the examined model presented some aspects of bone tissue deterioration, such as the decrease in cellular activity and the hypoxia-related effects, as well as a tendency for decreased Runx2, collagen type 1 and osteopontin expression. However, the conditions generated by the RPM also appear to support early osteogenic differentiation of MG-63 cells to a degree, as shown in the slight increase in ALP and fibronectin expression and a significant increase in ALP activity. To fully understand the microgravity effects within an Earth-based model, it would be advantageous to compare these findings with results from a similar study conducted in real microgravity conditions. Additionally, investigating various scaffold materials using the same microgravity-simulating device could help identify potential overlapping effects between the device and the materials on cellular responses. To further characterize the model, future studies should incorporate approaches such as omics analyses and whole-construct 3D imaging to provide a more comprehensive understanding of how RPM conditions influence molecular pathways, structural organization, and cell migration.

## Funding

This study was supported by the Program ‘Excellence Initiative – Research University’ and the subsidy (No. 16.16.160.557) for the AGH University of Krakow, Kraków, Poland, and the “Direction: Space” program, organized by the “New Space” Foundation and the “Empiria i Wiedza” Foundation. The authors thank Prof. Jacek Tarasiuk (AGH University of Krakow) for performing X-ray computed nanotomography characterization of the scaffolds.

## CRediT authorship contribution statement

**Barbara Szaflarska:** Writing – original draft, Visualization, Software, Methodology, Investigation, Data curation, Conceptualization. **Kamila Walczak:** Writing – review & editing, Validation, Software, Methodology, Investigation, Formal analysis, Data curation. **Marcin Czepiel:** Writing – review & editing, Methodology, Investigation. **Agata Kołodziejczyk:** Methodology. **Elżbieta Pamuła:** Writing – review & editing, Validation, Supervision, Resources, Project administration, Methodology, Funding acquisition, Formal analysis, Conceptualization.

## Declaration of competing interest

The authors declare that they have no known competing financial interests or personal relationships that could have appeared to influence the work reported in this paper.

## Data Availability

Data will be made available on request.

## Appendix Supplementary materials

Image, application 1
