# Supplementary material for: The influence of simulated microgravity on MG-63 osteoblast-like cells cultured on polymeric scaffold
Source: Biomater Biosyst. 2026 Apr 17;22:100137. doi: 10.1016/j.bbiosy.2026.100137 (PMC13112262; doi:10.1016/j.bbiosy.2026.100137)
Supplement: Supplementary file 1 [file mmc1.docx]

**Supplementary materials**

**The influence of simulated microgravity on MG-63 osteoblast-like cells cultured on polymeric scaffold**

**Barbara Szaflarska^1#^, Kamila Walczak^2#^, Marcin Czepiel^3^, Agata Kołodziejczyk^1,4^, Elżbieta Pamuła^2*^**

*^1^ AGH University of Kraków, Faculty of Space Technologies, al. Mickiewicza 30, 30-059 Kraków, Poland*

*^2^ AGH University of Kraków, Faculty of Materials Science and Ceramics, al. Mickiewicza 30, 30-059 Kraków, Poland*

*^3^ Jagiellonian University Medical College, Institute of Pediatrics, Department of Clinical Immunology , ul. Wielicka 265, 30-663 Kraków, Poland*

*^4^ Analog Astronaut Training Center, Koło Strzelnicy 8A, 30-219 Kraków, Poland*

* Corresponding author, email: epamula@agh.edu.pl

^#^ contributed equally to the study

Table 1S. Summary of the parameters changed between the preliminary Experiment 1 and optimized Experiment 2.

| **Experiment 1 (preliminary)** | **Experiment 2 (optimized)** | **Explanation** |
| --- | --- | --- |
| Scaffold diameter: 12mm | Scaffold diameter: 8.5 mm | Scaffold size and cell seeding density was lowered to increase the accuracy of microgravity simulation by enabling the use of smaller vials and therefore decreasing the residual accelerations exerted on the samples. The ratios between the scaffold mass, cell density, and medium volume remained the same in both experiments, so the impact on the results should be negligible. |
| No HEPES | HEPES added to media | HEPES was added to the media to optimize the pH in the enclosed vials. One of the purposes of the preliminary experiment 1 was to determine whether the vials can maintain the pH properly and as there were some changes observed, HEPES was added in the second experiment. To avoid introducing variability, it was added to samples in all conditions, not only the enclosed ones. |
| Collagen coating | No collagen coating | Collagen was used in the preliminary experiment to ensure proper adhesion of the cells to the scaffold, however, there were no differences observed in the morphology or the number of cells populating the coated and non-coated scaffolds, as shown by imaging. Furthermore, as the goal of the study was to create a simple, reproducible model, it was decided not to use collagen, as it can introduce great variability depending on its source. |
| Rotation speed: 60 rpm | Rotation speed: 10 rpm | As the preliminary experiment was performed to test the designed protocols, the factory setting of ‘micro-g’ was left unchanged. In the second experiment, upon the literature search, the speed of rotation was changed to match the current knowledge. |

Table 2S. Primer sequences used in the RT-qPCR in Experiment 2

| Gene | Forward Primer | Reverse Primer |
| --- | --- | --- |
| Fib1 | CTGAGTACACCGTATCCCTCG | CCAGGCTGCAGTGTGGTA |
| Runx2 | AGGACAGCAAGAAGTCTCTGG | CTGCTTGCAGCCTTAAATGACT |
| ALP | CCGAGATACAAGCACTCCCA | CTGGCTCGAAGAGACCCAAT |
| Col1 | AGTGTGGCCCAGAAGAACTG | CCGCCATACTCGAACTGGAA |
| SPP1 | ACCTGACATCCAGTACCCTGA | AACGGGGATGGCCTTGTATG |
| BGLAP | ATGAGAGCCCTCACACTCCT | CTTGGACACAAAGGCTGCAC |
| GAPDH | GGCATGGACTGTGGTCATGAG | CTGCACCACCAACTGCTTAGC |


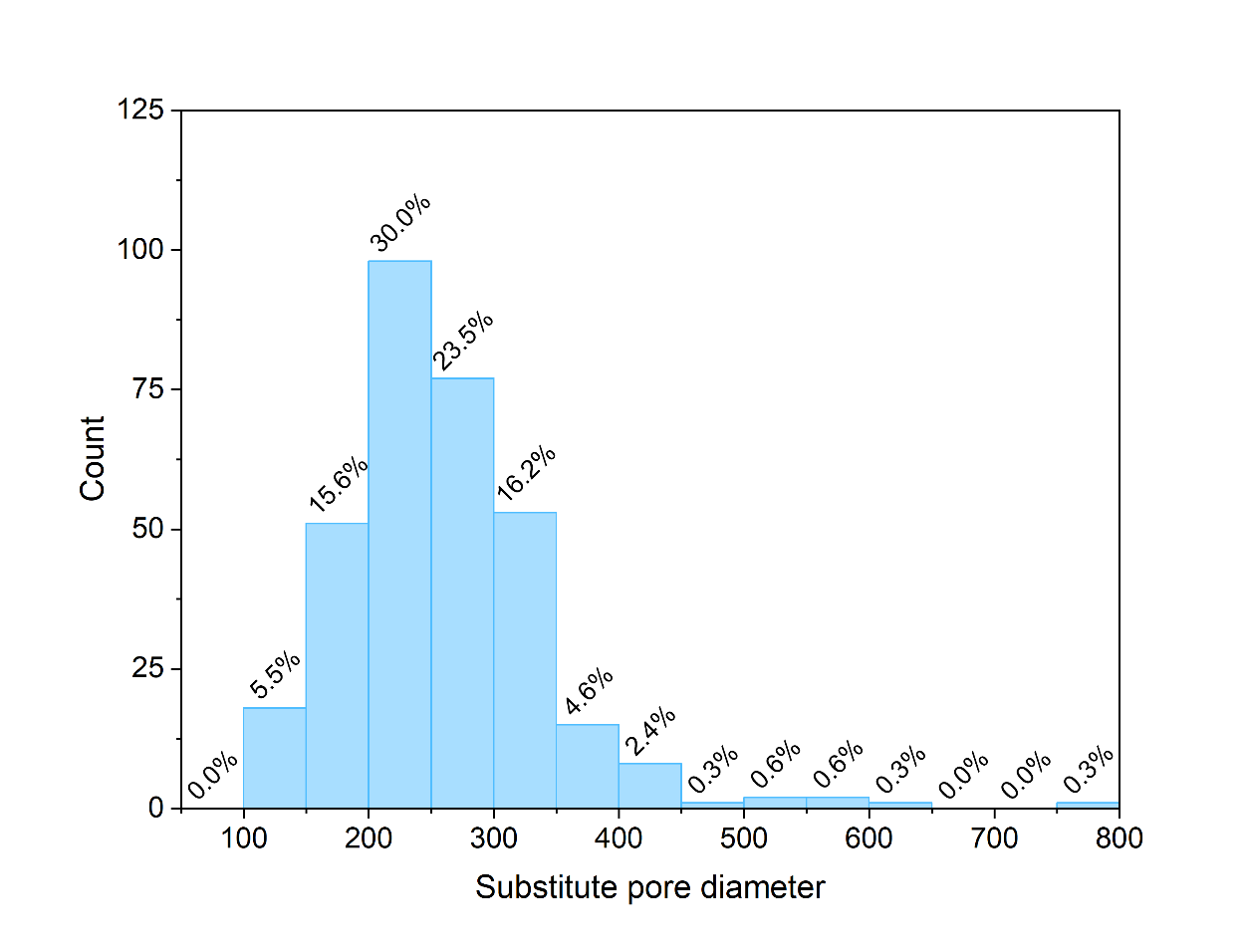


Figure 1S. Histogram of the pore size of the scaffolds
